# Advanced Machining Technology for Modern Engineering Materials

**DOI:** 10.3390/ma17092064

**Published:** 2024-04-28

**Authors:** Panagiotis Karmiris-Obratanski, Muthuramalingam Thangaraj, Beata Leszczyńska-Madej, Angelos P. Markopoulos

**Affiliations:** 1 Advanced Manufacturing Laboratory, Department of Manufacturing Systems, Faculty of Mechanical Engineering and Robotics, AGH University of Krakow, 30-059 Krakow, Poland; 2 Department of Mechatronics Engineering, SRM Institute of Science and Technology, SRM Nagar, Chennai 603203, TN, India; muthurat@srmist.edu.in; 3 Faculty of Non Ferrous Metals, Department of Materials Science and Non-Ferrous Metals Engineering, AGH University of Krakow, Mickiewicza 30 Av., 30-059 Krakow, Poland; bleszcz@agh.edu.pl; 4 Laboratory of Manufacturing Technology, School of Mechanical Engineering, National Technical University of Athens, 10682 Athens, Greece; amark@mail.ntua.gr

Advances in material science have indeed revolutionized engineering, bringing forth a suite of new materials with remarkable properties. Superalloys, known for their exceptional heat resistance and strength, are now crucial in high-temperature applications such as jet engines and industrial gas turbines. Memory alloys, which can revert to their original shape after deformation, have found widespread use in medical devices and actuators. Advanced composite materials, combining strength and lightness, have transformed the aerospace and automotive industries, while biocompatible materials have opened new horizons in medical implants and prosthetics.

The machining industry, traditionally equipped to handle conventional materials like steel and aluminum, now faces the significant challenge of adapting to these new materials. Each of these advanced materials presents unique machining difficulties. For instance, the high strength and temperature resistance of superalloys make them challenging to cut or shape using standard tools and techniques. Memory alloys’ ability to change shape can complicate machining processes that rely on material rigidity. Similarly, the heterogeneous nature of composite materials often leads to issues like delamination or fiber pull-out during machining. To address these challenges, the industry is gravitating towards innovative machining methods and processes. Non-conventional machining processes, such as electrical discharge machining (EDM), laser machining, and ultrasonic machining, are increasingly being employed to handle the complexities of these materials. These methods offer the advantages of precision and minimized mechanical stresses during the machining process.

Moreover, the development of hybrid machining processes, combining conventional and non-conventional techniques, is a growing area of interest. These processes harness the benefits of both methods, such as the efficiency of traditional machining and precision of modern techniques. For instance, laser-assisted machining (LAM) combines a laser’s thermal energy with mechanical cutting to facilitate the machining of tough superalloys.

This Special Issue represented a call to action for the global research community, inviting contributions that highlight the forefront of scientific inquiry and innovation in the realm of advanced machining technologies. It curates a comprehensive collection of scholarly works elucidating the latest technological advancements and methodological breakthroughs in this field. Submissions to this Special Issue will not only encapsulate the current state of non-conventional precision machining processes but also pave the way for future scientific exploration and development trends within this critical and dynamically evolving field of study.

In this Special Issue, the initial study focuses on comprehensive design theory for involute splines made of PEEK (polyetheretherketone), enhancing their performance and refine the design rules for such components. The theory integrates international (ISO 4156, 1. 2. 3, 2005) and American standards (ANSI B92.2M. 1989) with traditional empirical formulas. A key innovation of the study is the adaptation of the involute splines calibration method, rooted in international standards, to suit PEEK-based materials. This adaptation involves estimating the effects of viscoelastic energy consumption on temperature field calibration. The authors found that viscoelasticity is crucial for PEEK spline temperature calibration, enhancing reliability. Better stress was detected at a 45° angle, with modulus and length affecting the stress uniformity [1]. The second contribution of Li, Zheng, and Feng [2] optimizes cutting parameters for machining deep bottle holes using cutting simulation, regression analysis genetic algorithms, and experimental validation. It analyzes the impacts of these parameters on the cutting force and temperature using the Response Surface Method, establishing a regression prediction model. Optimal parameters (139.41 m/min speed, 1.12 mm depth, 0.27 mm/rev feed) were identified for low force and high removal rate. Each parameter’s effectiveness was confirmed by machining TC4 deep holes, providing a reference for similar machining tasks. On the other hand, besides investigating machinability, it is essential to examine the durability of components against fatigue cracking. Therefore, the third contribution in this Special Issue focuses on conducting in-depth research into the effects of forging conditions on the surface and microstructure of tool steel. This research could lead to the development of optimal tool materials characterized by enhanced durability and resistance to wear, significantly improving the lifespans and efficacies of forging tools. This study addresses a gap in our current knowledge of the behavior of forging tool steel [3]. The analysis of 3D scans revealed that material loss, abrasive wear, and plastic deformation affect tool geometry, in turn influencing the formation of fatigue cracks. In case of AM, the Inter-Layer Cooling Time (ILCT) affects the microstructures of the components. Indeed, in the fourth contribution, the authors tried to determine the critical ILCT range for optimal printing, examining how changes in ILCT, ranging from 22 to 2 s, affect material porosity and melt pool characteristics in printed cylinder specimens. Their findings indicate that ILCT values below 6 s significantly alter the material’s microstructure, particularly at 2 s, where increased keyhole porosities and deeper melt pool depths are observed. This finding recommends making changes to the powder melting regime and printability window [4].

The fifth contribution analyzes bonding in friction stir spot welding using finite element analysis and optimizes parameters through artificial neural networks. It focuses on pressure–time and pressure–time–flow criteria, identifying the latter as more suitable for FSSW. The experimental results are compared with the predictions: for bonding strength, the experimental value was 4.0 kN, closely matching the predicted value of 4.147 kN, with a minimal error of 3.675%. For hardness, the experimental value was 62 Hv compared to the predicted value of 60.018 Hv, showing an error of 3.197%. This comparison confirms the effectiveness of the optimized parameters and the reliability of the artificial neural network predictions [5]. The sixth contribution addresses the gap between infill density and printing speed in 3D-printed acrylonitrile butadiene styrene (ABS) [6]. The authors, using a full central composite design (CCD) and ANOVA, determine the optimal 3D printer settings to balance conflicting responses such as flexural strength (FS), tensile strength (TS), average surface roughness (Ra), print time (T), and energy consumption (E). Through numerical multi-objective optimization, the study identifies optimal parameters: a layer thickness (LT) of 0.27 mm, an infill density (ID) of 84%, and a printing speed (PS) of 51.1 mm/s. These settings resulted in an FS of 58.01 MPa, a TS of 35.8 MPa, the lowest possible Ra of 8.01 µm, the lowest possible T of 58 min, and an E of 0.21 kWh. The next study determines the impact of varying the input power of a diode laser on key aspects of the cutting process, including carbonization, kerf width, and the material removal rate (MRR). To achieve this, a diode-based laser beam machining setup was developed, featuring diode lasers with power ratings of 2.5 W, 5.5 W, and 20 W. A notable finding is that a high-power laser with a smaller spot size in pulsed mode can create a higher power density, which reduces the duration of the interaction with the material, thus minimizing carbonization. Furthermore, the 20 W diode laser’s ability to control beam shape and size enables it to achieve a narrower kerf width and higher MRR. The study also identifies the optimal parameters for cutting chrome vegetable tanned buffalo leather: a standoff distance of 18 mm, a feed rate of 200 mm/min, and a duty cycle of 70%. These findings provide valuable insights for improving the efficiency and quality of leather cutting processes in various industries [7].

Cutting fluids enhance machining by reducing heat and friction, thus improving tool life, surface finish, and chip removal. Consequently, the next two articles (Contributions [8,9]) analyze the effects of cutting fluids on machining performance and machinability. Contribution 8 examines the impacts of various cutting fluids on critical aspects of milled surfaces, including surface roughness, morphology, residual stress, and elemental composition. This study’s unique focus on the properties of the cutting fluids themselves and their relation to the quality and integrity of machined surfaces is a standout feature. The findings revealed that among water and E709, the Blasocut fluid shows better lubrication, lower roughness, and smaller residual stresses. However, the Blasocut fluid also slows down surface corrosion, while E709 tends to lead to stable pitting corrosion over time [8]. On the other hand, the ninth contribution optimizes helical milling parameters to improve quality and reduce tool wear by using MoS_2_-based MQL for machining CFRP/Ti stacks, focusing on surface integrity and temperature. The findings show that spindle speed, tangential feed, and eccentricity are crucial for determining hole accuracy and surface roughness in CFRP/Ti milling. The MoS_2_ concentration in MQL is key for temperature control, affecting tool wear [9]. In contrast to previous studies, the tenth contribution in this series designed and applied a Real-Time Extremum-Seeking Controller (ESC) to optimize the material removal rate (MRR) through low-power electric discharge machining (EDM). The ESC, when integrated into the EDM process’s feedback loop, addresses the non-linear relationship between the MRR and discharge time. This method aligns more closely with the actual process when tested with steel workpieces and copper electrodes, having a lower error rate compared to traditional experimental approaches. The results indicate that the ESC controller achieves a 57% greater material removal rate than the average of manual trial processes and is 1.2% more efficient than the best manual search process [10]. The subsequent contribution utilizes advanced machine learning algorithms to model and optimize the Equal Channel Angular Pressing (ECAP) process for copper. This approach improves material properties while reducing the need for extensive experimental work, representing significant progress in the development of material design processes. The study’s key findings indicate that the 90° die is more effective than the 120° die, as well as that as-annealed parts could achieve an up to a 72% increase in hardness [11].

The final two contributions focus primarily on improving the machining of hard-to-cut materials using traditional techniques. The twelve contribution employs 3D FE models to assess the performances of self-propelled rotary tools (SPRTs) in hard turning, predicting chip morphology, cutting forces, and stress/temperature dynamics. Experimental and simulation results indicate that lower feed rates, resulting in higher chip flow angles, lead to chip curling, as well as that the cutting force and von Mises stress fluctuate with the tool inclination angle, with reduced von Mises stress identified at higher angles [12]. Lastly, the study [13] uses Deform-3D to model both ordinary and vibration drilling of Inconel-718, demonstrating that vibration drilling effectively reduces the temperatures of both tool teeth (by up to 21.1%) and workpiece (by 5.7%). These findings contribute to enhanced cutting efficiency and prolonged operational lifespan in the drilling of nickel-based alloys.

The Guest Editors of this Special Issue extend their gratitude to the authors for their significant and high-quality contributions, the reviewers for dedicating effort and time for refining the submissions, and the publisher for their outstanding work and collaboration.

## References

[1] Xue X., Yu W., Lin K., Zhang N., Xiao L., Jiang Y. (2024). Design Method and Teeth Contact Simulation of PEEK Involute Spline Couplings. Materials.

[2] Li W., Zheng H., Feng Y. (2023). Optimization of Cutting Parameters for Deep Hole Boring of Ti-6Al-4V Deep Bottle Hole. Materials.

[3] Hawryluk M., Lachowicz M., Łukaszek-Sołek A., Lisiecki Ł., Ficak G., Cygan P. (2023). Structural Features of Fatigue Crack Propagation of a Forging Die Made of Chromium–Molybdenum–Vanadium Tool Steel on Its Durability. Materials.

[4] Baldi N., Giorgetti A., Palladino M., Giovannetti I., Arcidiacono G., Citti P. (2023). Study on the Effect of Inter-Layer Cooling Time on Porosity and Melt Pool in Inconel 718 Components Processed by Laser Powder Bed Fusion. Materials.

[5] Jo D.S., Kahhal P., Kim J.H. (2023). Optimization of Friction Stir Spot Welding Process Using Bonding Criterion and Artificial Neural Network. Materials.

[6] Mushtaq R.T., Iqbal A., Wang Y., Rehman M., Petra M.I. (2023). Investigation and Optimization of Effects of 3D Printer Process Parameters on Performance Parameters. Materials.

[7] Khalaf T., Thangaraj M., Moiduddin K., Swaminathan V., Mian S.H., Ahmed F., Aboudaif M.K. (2023). Performance Evaluation of Input Power of Diode Laser on Machined Leather Specimen in Laser Beam Cutting Process. Materials.

[8] Chen S., Yan P., Zhu J., Wang Y., Zhao W., Jiao L., Wang X. (2023). Effect of Cutting Fluid on Machined Surface Integrity and Corrosion Property of Nickel Based Superalloy. Materials.

[9] Mughal K., Mughal M.P., Farooq M.U., Qaiser Saleem M., Haber Guerra R. (2023). Helical Milling of CFRP/Ti6Al4V Stacks Using Nano Fluid Based Minimum Quantity Lubrication (NF-MQL): Investigations on Process Performance and Hole Integrity. Materials.

[10] Ismail M.R.M., Thangaraj M., Karmiris-Obratański P., Papazoglou E., Karkalos N. (2023). Design of Real-Time Extremum-Seeking Controller-Based Modelling for Optimizing MRR in Low Power EDM. Materials.

[11] Shaban M., Alsharekh M.F., Alsunaydih F.N., Alateyah A.I., Alawad M.O., BaQais A., Kamel M., Nassef A., El-Hadek M.A., El-Garaihy W.H. (2022). Investigation of the Effect of ECAP Parameters on Hardness, Tensile Properties, Impact Toughness, and Electrical Conductivity of Pure Cu through Machine Learning Predictive Models. Materials.

[12] Umer U., Mian S.H., Mohammed M.K., Abidi M.H., Moiduddin K., Kishawy H. (2022). Self-Propelled Rotary Tools in Hard Turning: Analysis and Optimization via Finite Element Models. Materials.

[13] Shi Y., Zheng J., Feng P., Shang P., Liu C., Chen T., Shan S. (2022). BTA Deep Hole Vibration Drilling for Nickel-Based Alloys: Cooling Patterns and Cutter Tooth Wear Mechanisms. Materials.

